# Ni(0)-CMC-Na Nickel Colloids in Sodium Carboxymethyl-Cellulose: Catalytic Evaluation in Hydrogenation Reactions

**DOI:** 10.3390/molecules16010367

**Published:** 2011-01-07

**Authors:** Mohamed Anouar Harrad, Pedro Valerga, M. Carmen Puerta, Issam Houssini, Mustapha Ait Ali, Larbi El Firdoussi, Abdallah Karim

**Affiliations:** 1 Coordination Chemistry and Catalysis Team, Department of Chemistry, Faculty of Science, Semlalia, Cadi Ayyad University, BP 2390, 40001 Marrakech, Morocco; 2 Department of Materials Science, Metallurgical Engineering and Inorganic Chemistry, Faculty of Science, Campus Rıo San Pedro, Puerto Real 11510, Spain

**Keywords:** colloids, hydrogenation, bioorganic polymer

## Abstract

A recyclable catalyst, Ni(0)-CMC-Na, composed of nickel colloids dispersed in a water soluble bioorganic polymer, sodium carboxymethylcellulose (CMC-Na), was synthesized by a simple procedure from readily available reagents. The catalyst thus obtained is stable and highly active in alkene hydrogenations.

## 1. Introduction

In recent years, metal colloids have fascinated scientists because of their outstanding electronic and optical properties [1,2,3]. They have become important catalysis research candidates [4], used in a wide variety of hydrosilylation [5], oxidation [6], C-C coupling [7,8] and selective hydrogenation reactions [9,10,11]. Their potential utilities are evaluated in terms of activity, selectivity, physical and chemical stability [3]. Colloids have major applications in heterogeneous catalysis [12] due to their high specific surface area. The organometallic approach followed in the laboratory for the synthesis of colloids provides access to various metal colloids (Pd, Rh, Ru…) in a controlled and reproducible way, but it would be of interest to develop other types of colloidal catalyst using less expensive transition metals, such as nickel. Our work was focused on the synthesis of a new colloidal species based on nickel, stabilized by a water soluble bioorganic polymer, sodium carboxymethylcellulose (CMC-Na). The colloids obtained were used as catalysts in the hydrogenation of functionalized olefins. With the aim of optimizing the operating conditions, we have studied different parameters influencing the course of the reaction such as the molar ratio substrate/catalyst (S/C), nature of solvent, polymer concentration and the nature of the substrate.

## 2. Results and Discussion

The nickel(0) colloids were generated from nickel(II) chloride, sodium borohydride and a catalytic amount of sodium carboxymethylcellulose (CMC-Na) in a mixture of water/methanol at room temperature. The prepared material was characterized by X-ray diffraction according to literature procedures [13,14,15,16,17].

**Figure 1:**
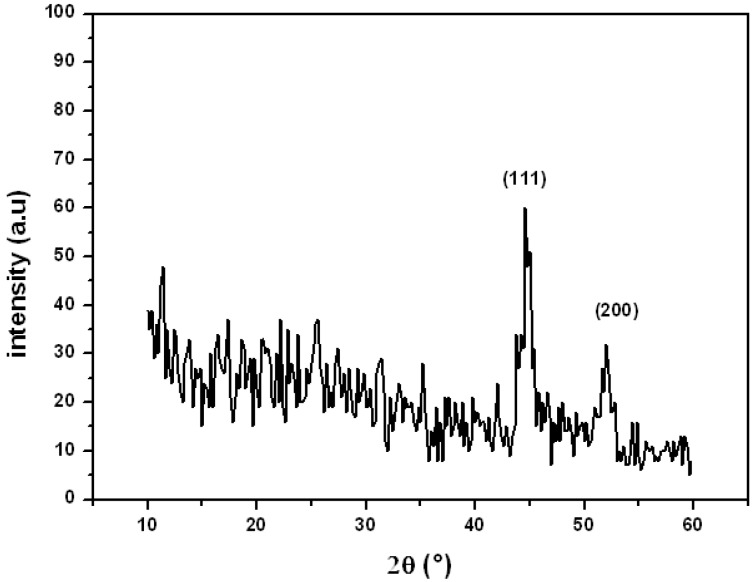
XRD patterns of the preparednickel colloid.

Figure 1 shows the typical X-ray diffraction pattern of the Ni-CMCNa nanoparticles. Due to the small size effect and incomplete inner structure of the particle, the XRD peaks are low and broad. On the other hand, the XRD pattern shows that the sample is two-phase. The peaks with 2θ values around 44.5° and 53° correspond to the (111) and (200) planes of the metallic Ni phase respectively, which is in accordance with that of the standard spectrum (JCPDS, No. 88-2326) [13,14,15,16,17].

In order to optimize the hydrogenation conditions, a preliminary study was carried out using cinnamyl alcohol (**1**) as a model substrate. The reaction was conducted at room temperature, using different solvents, various concentrations of CMC-Na polymer and under different hydrogen pressures (Table 1). The hydrogenation of **1**, in aqueous solution under one atmosphere of hydrogen, yielded dihydrocinnamyl alcohol (**2**) in moderate yield after 7 h (entry 1, Table 1). A combination of water and alcohol as solvent is necessary to increase the activity of catalyst, and the best results were obtained when mixtures of water and methanol or water and isopropanol were used as solvent (entries 2 and 7). Increasing the hydrogen pressure has a favorable effect on the catalyst activity and minimizes the reaction time considerably (entries 8, 9 and 10). However, increasing the polymer concentration is unfavourable for the catalytic activity. When more than 0.5 g/L of CMC-Na is used, the conversion decreases considerably (entries 11-14). In all cases, dihydrocinnamyl alcohol **2** was the sole product obtained with 100% selectivity.

**Table 1:** Hydrogenation of cinnamyl alcohol (**1**) catalyzed by Ni(0)-CMC-Na colloids: Influence of nature of solvent, concentration of polymer and hydrogen pressure. 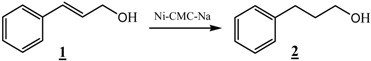

Entry	Polymer concentration g/L	Solvent	H_2_ pressure (bars)	Time (h)	Conversion %
**1**	0.5	H_2_O	1	7	45
**2**	0.5	H_2_O/iPrOH	1	7	87
**3**	0.5	H_2_O/CHCl_3_	1	24	5
**4**	0.5	H_2_O/THF	1	12	2
**5**	0.5	H_2_O/Acetonitril	1	16	5
**6**	0.5	H_2_O/toluene	1	24	0
**7**	0.5	H_2_O/MeOH	1	7	90
**8**	0.5	H_2_O/MeOH	10	5	91
**9**	0.5	H_2_O/MeOH	20	3.5	92
**10**	0.5	H_2_O/MeOH	40	2.0	98
**11**	1.0	H_2_O/MeOH	1	7	62
**12**	1.5	H_2_O/MeOH	1	7	57,5
**13**	2.0	H_2_O/MeOH	1	7	40
**14**	3.0	H_2_O/MeOH	1	7	12

Reaction conditions: S/C = 100, 10 mL of solvent (8/2), room temperature. ^a^ The conversions were determined by gas chromatography.

We note here that the recyclability of the catalyst was verified by submitting the same recovered Ni colloid catalyst to three subsequent reaction cycles. No appreciable loss in activity was observed before two cycles, as shown in Table 2. 

**Table 2:** Hydrogenation of cinnamyl alcohol catalyzed by Ni(0)-CMC-Na colloids: catalyst recyclability.

Entry	Reuses	Conversion %
**7**	0	90
**15**	1	81
**16**	2	63
**17**	3	40

Reaction conditions: S/C = 100, 10 mL of solvent: H_2_O/MeOH (8/2), room temperature, 7 h. ^a^ The conversion were determined by gas chromatography.

Reduction of other functionalized olefins: 1,3-diphenylpropenone (**3**), 4-phenylbut-3-en-2-one (**4**), 5-allylbenzo[1,3]dioxole (**5**) and 5-propenylbenzo[1,3]dioxole (**6**) under identical conditions using Ni(0)-CMC-Na colloid catalyst led to the corresponding products in good to excellent yields (60–90%) (Table 3). In all cases these good yields were achieved using low hydrogen pressure, at room temperature and after acceptable reaction times. We note here that only the double band was hydrogenated with 100% selectivity and other functional groups remain intact.

**Table 3:** Hydrogenation of olefins catalyzed by Ni(0)-CMC-Na colloid.

Entry	Substrate	Product	Time (h)	Conversion %	Isolated yield %
**18**	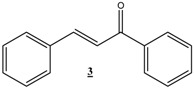	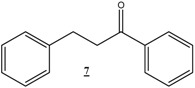	48	72	80
**19**	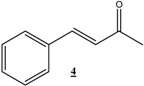	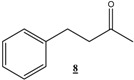	48	86	63
**20**	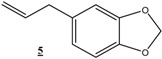	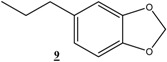	36	100	88
**21**	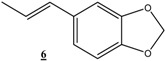	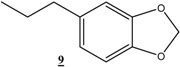	48	93	79

Conditions: S/C=100, 0.5 g/L CMC-Na, solvent: MeOH/H_2_O (8/2), P H_2_: 40 bars, room temperature. ^a^ The conversions were determined by gas chromatography.

## 3. Experimental

### 3.1. Instrumentation

NMR studies were performed on a Bruker Avance 300 spectrometer in CDCl_3_. Chemicals shifts are given in ppm relative to external TMS and coupling constants (*J*) in Hz, Mass spectra were recorded on a GC-MS Thermofinnigan Polaris-Q mass spectrometer. The synthesized nanoparticles were characterized by wide angle X-ray diffraction (Philips XPERT-MPD diffractometer, Goniometer = PW3050/10 with Cu(0) anode material). 

### 3.2. Preparation of the Metal Colloid Precursors

Sodium borohydride (5 mg) was added to an aqueous solution of CMC-Na surfactant (5 mg, in 40 mL H_2_O). The obtained solution was quickly added under vigorous magnetic stirring to an aqueous solution of the precursor NiCl_2_6H_2_O (50 mg, 0.42 mmol) in H_2_O (10 mL). The initial green solution darkened immediately and after one night, the suspensions obtained remain stable for a week under nitrogen.

### 3.3. General Hydrogenation Procedure

A round-bottom flask (25 mL), charged with the aqueous suspension of Ni(0)-CMC-Na (10 mL) and a magnetic stirrer, was connected to a gas burette (500 mL) with a flask to balance the pressure. The flask was closed with a septum and the system filled with hydrogen. The appropriate substrate (S/C = 100) was injected through a septum and the mixture was stirred. The reaction was monitored by volume of gas consumed and by gas chromatography (Varian 3800 equipped with a RTX5 capillary column and a FID detector). At the end of the reaction, the two phases were separated and the aqueous phase was extracted with ether (3 × 25 mL). The combined ether solution was dried over anhydrous Na_2_SO_4_. The solvent was removed under reduced pressure and the residue was purified by column chromatography over silica gel.

### 3.4. Typical Spectral Data

*1,3-Diphenyl-propan-1-one* (**7**): ^1^H-NMR (CDCl_3_, 300 MHz) δ: 1.98 (t, 2H), 2.2 (t, 2H), 7.2-7.94 (m, 10H); ^13^C-NMR (CDCl_3_, 75 MHz) δ: 33.14, 46.70, 122.03, 126.18, 128.11, 46, 128.71, 129.25, 130.50, 132.95, 135.05, 138.34, 144.16, 203.12.

*4-Phenylbutan-2-one* (**8**): ^1^H-NMR (CDCl_3_, 300 MHz) δ: 1.63 (s, 3H), 1.92 (t, 2H), 2.09 (t, 2H), 7-7.2 (m, 5H); ^13^C-NMR (CDCl_3_, 75 MHz) δ: 30.03, 32.14, 45.17, 125.43, 125.81, 126.46, 127.29, 128.71, 140.16, 205.8.

*5-Propylbenzo[1,3]dioxole* (**9**): ^1^H-NMR (CDCl_3_, 300 MHz) δ: 1.16 (t, 3H), 1.8 (m, 2H), 2.7 (t, 2H), 6.13 (s, 2H), 8.8-6.95. (m, 3H); ^13^C-NMR (CDCl_3_, 75 MHz) δ: 13.6, 24.5, 37.73, 100.44, 107.86, 108.79, 120.71, 136.16, 145.4, 147.37.

## 4. Conclusions

Nickel(0) colloids prepared at room temperature from nickel(II) chloride, sodium borohydride and sodium carboxy-methylcellulose (CMC-Na) in a mixture of water/methanol, have shown to promote the ready hydrogenation of a variety of functionalized olefins at room temperature and moderate hydrogen pressures. Moreover, the nickel colloids could be re-utilized several times, maintaining a moderate to high activity.
